# Comparison of rectal dose reduction by a hydrogel spacer among 3D conformal radiotherapy, volumetric-modulated arc therapy, helical tomotherapy, CyberKnife and proton therapy

**DOI:** 10.1093/jrr/rraa013

**Published:** 2020-03-25

**Authors:** Masahide Saito, Toshihiro Suzuki, Yuya Sugama, Kan Marino, Naoki Sano, Takafumi Komiyama, Shinichi Aoki, Yoshiyasu Maehata, Kazuya Yoshizawa, Kazunari Ashizawa, Hidekazu Suzuki, Koji Ueda, Yosuke Miyasaka, Masayuki Araya, Hiroshi Takahashi, Hiroshi Onishi

**Affiliations:** 1 Department of Radiology, University of Yamanashi, Yamanashi, Japan; 2 Kasugai CyberKnife Rehabilitation Hospital, Yamanashi, Japan; 3 Aizawa Hospital Proton Therapy Center, Matsumoto, Japan

**Keywords:** hydrogel spacer, prostate cancer, rectum dose, CyberKnife, proton therapy

## Abstract

This study aimed to evaluate the rectal dose reduction with hydrogel spacer in 3D conformal radiotherapy (3DCRT), volumetric modulated arc therapy (VMAT), helical tomotherapy (HT), CyberKnife (CK) and proton therapy. Twenty patients who had hydrogel spacer for prostate radiotherapy were retrospectively enrolled. Computed tomography (CT) images with or without hydrogel spacer were used to evaluate rectal dose reduction. In total, 200 plans (20 patients × 2 CT images × 5 techniques) were created using the following criteria: 3DCRT, VMAT and HT [76 Gy/38 fractions (Fr), planning target volume (PTV) D50%], CK (36.25 Gy/5 Fr, PTV D95%) and proton therapy (63 GyE/21 Fr, PTV D50%). Rectal dose reduction was evaluated using low-/middle-dose (D20%, D50% and D80%) and high-dose (D2%) ranges. Rectal dose reduction of each dose index was compared for each technique. Significant rectal dose reduction (*P* < 0.001) between the treatment plans on pre- and post-CT images were achieved for all modalities for D50%, D20% and D2%. In particular, the dose reduction of high-dose (D2%) ranges were −40.61 ± 11.19, −32.44 ± 5.51, −25.90 ± 9.89, −13.63 ± 8.27 and −8.06 ± 4.19%, for proton therapy, CK, HT, VMAT and 3DCRT, respectively. The area under the rectum dose–volume histogram curves were 34.15 ± 3.67 and 34.36 ± 5.24% (*P* = 0.7841) for 3DCRT with hydrogel spacer and VMAT without hydrogel spacer, respectively. Our results indicated that 3DCRT with hydrogel spacer would reduce the medical cost by replacing the conventional VMAT without spacer for prostate cancer treatment, from the point of view of the rectal dose. For the high-dose gradient region, proton therapy and SBRT with CK showed larger rectal dose reduction than other techniques.

## INTRODUCTION

A high conformal external beam radiotherapy (EBRT) is conventionally used to treat patients with low or intermediate risk for prostate cancer and shows favorable treatment outcomes compared with surgery [1]. Currently, many EBRT techniques are available, such as 3D conformal radiotherapy (3DCRT), intensity-modulated radiotherapy (IMRT), volumetric modulated arc radiotherapy (VMAT), stereotactic body radiotherapy (SBRT) and proton therapy. Although dose escalation with these techniques leads to a better treatment outcome [2, 3], toxicities on the rectum are still an important problem especially with 3DCRT [4]. As one of the solutions to reduce rectal dose, a hydrogel spacer (SpaceOAR™ System, Augmenix Inc., Waltham, MA) has been introduced and used for EBRT in prostate cancer patients. A phase 3 trial for image-guided IMRT shows the benefit of a hydrogel spacer in reducing rectal dose, toxicity and quality of life decline [5].

A number of studies reported the effectiveness of treatment planning using the hydrogel spacer [6–8]. Although the effect of rectal dose reduction is well-known, the characteristics of different EBRT techniques have not been evaluated in detail, and the difference in the effects of irradiation methods is unclear. In particular, one of our important hypotheses is that 3DCRT with hydrogel spacer might show comparable dose distribution with VMAT without hydrogel spacer. This hypothesis is important especially for some facilities where IMRT is not possible. Therefore, this study aimed to evaluate rectal dose reduction with hydrogel spacer in five types of EBRT techniques: 3DCRT, VMAT, helical tomotherapy (HT), CyberKnife (CK) and proton therapy.

## MATERIALS AND METHODS

### Study design

This retrospective study was approved by the institutional review board (IRB) of the University of Yamanashi. From July 2018 to March 2019, 20 patients who underwent prostate radiotherapy with a hydrogel spacer (SpaceOAR™ System, Augmenix Inc., Waltham, MA) were enrolled. In this study, the eligibility criteria for hydrogel spacer implantation were as follows: (i) case with SBRT, (ii) patients with a high risk of hemorrhage such as those with diabetes mellitus or receiving anticoagulants and (iii) patients with large prostatic volume near the rectum.

### Procedure

All patients were treated with SBRT [36.25 Gy/5 fractions (Fr), planning target volume (PTV) D95%] in CK or hypofractionated treatment with IMRT (62 Gy/20 Fr, PTV D50%) in HT following hydrogel implantation. The hydrogel implantation method has previously been described [6, 9]. In our institute, the spacer was inserted by radiation oncologists. In brief, the patient is positioned in the dorsal lithotomy position with a transrectal ultrasound probe positioned in the rectum. Then, a needle is subsequently advanced to the midpoint (base to apex) of the prostate gland, and the spacer is injected between the rectum and prostate under transrectal ultrasound guidance and a stepper device. The spacer solidifies in seconds and it maintains a space between the prostate and the rectum for ~3 months. Finally, the spacer is absorbed into the body over ~6 months.

The study was performed with the following procedure. First, computed tomography (CT) images at pre-SpaceOAR injection [SO(−)] were acquired for all patients. After the injection, CT images with the SpaceOAR [SO(+)] were acquired. Note that the condition of the patient setup for each image acquisition was slightly different because spacer placement took 30–60 min. After obtaining each CT image, five types of treatment planning were performed by three clinical medical physicists, and statistical analysis was performed.

### Contouring and treatment planning protocol

Three medical physicists contoured all organs at risk (OARs) and targets and created all plans. For OARs, the rectum [contoured ±5 CT slices from PTV (thickness of CT slice, 2 mm)], bladder and urethra were contoured. In a clinical setting, the clinical target volume (CTV) in our protocol was different between risk classifications: high risk, prostate plus 2 cm of the seminal vesicle for cases lower than T3b, or total seminal vesicle invasion with satisfactory margin for T3b cases; intermediate risk, CTV plus 1 cm of the base of the seminal vesicles; and low risk, prostate. However, only four high-risk patients were included in this study; therefore, all CTV was set as low risk to reduce treatment plan variability due to target-shape complexity. Additionally, the PTV margin setting and beam arrangement were different among the five techniques, which is explained as following section. The treatment plans were created for CT images of SO(−) and SO(+) for five irradiation techniques, respectively, i.e. 20 patients × 2 CT images × 5 irradiation techniques; thus, a total of 200 plans were created.

### 3DCRT

Treatment planning was performed by Pinnacle TPS (Philips Radiation Oncology Systems, Madison, WI)) using Elekta Synergy with Agility gantry head, which has 160 multi-leaf collimator leaves of 5 mm (Elekta AB, Stockholm, Sweden). Isocenter of the beams was located at the center of PTV. All patients received a dose covering 50% volume (D50%) prescription of 76 Gy for PTV in 38 Fr. The adaptive convolve in Pinnacle was used as the dose calculation algorithm, and the dose grid size was 2.5 mm for all dimensions. The PTV margin from the CTV was defined as 8 mm in the anterior and superior; 5 mm in the left, right and inferior; and 4 mm in the posterior directions. Five fixed beams (0, 75, 120, 240 and 285°) were used with 10 MV X-ray. For all beams, the collimator angle was 0° and a port margin was created by 5-mm PTV expansion. The doses of the rectum and bladder were reduced as much as possible.

### VMAT and HT

For VMAT, treatment planning was performed by Pinnacle TPS using the SmartArc optimization algorithm. Treatment machine, dose calculation algorithm, dose grid size and PTV margin setting were the same as in the aforementioned 3DCRT conditions. Beam energy was 10 MV, and one full arc (181 to 179° clockwise) with a collimator angle of 15° was used. All patients received a dose covering 50% volume (D50%) prescription of 76 Gy for PTV in 38 Fr. The dose constraints of the rectum were D2% < 74.4 Gy, D10% < 66.5 Gy and D50% < 20.0 Gy. The dose constraints of the bladder were D15% < 73 Gy, D25% < 70 Gy, D35% < 67 Gy and D50% < 57 Gy.

For HT, treatment planning was performed by Tomotherapy Planning Station (Accuray, Sunnyvale, CA) and TomoHD (Accuray, Sunnyvale, CA) was used as the treatment machine. The plans for HT were created with a dynamic jaw mode using a jaw size of 2.51 cm and pitch size of 0.287 cm in all cases. The convolutional/superposition algorithm with the fine dose grid was used as the dose calculation algorithm. The dose prescription and dose constraints were the same as those in VMAT.

### CK

The SBRT plan was created for all patients using CyberKnife MultiPlan TPS (Accuray, Sunnyvale, CA) with a sequential optimization algorithm. CyberKnife G4 (Accuray, Sunnyvale, CA) with Fixed and Iris collimator was used as the treatment machine. All plans typically used 2–3 fixed collimators of different sizes and 150–300 noncoplanar and non-isocentric beams of 6 MV. The prescription dose was calculated using the Monte Carlo algorithm with a 2% uncertainty on a 1 × 1 × 1.25-mm resolution. The PTV margin from the CTV was defined as 3 mm in the posterior direction and 5 mm in other directions. All patients received a dose covering 95% volume (D95%) prescription of 36.25 Gy for PTV in 5 Fr. The dose constraints of the rectum and bladder were D1cc < 35.0 Gy and D1cc < 39.0 Gy, respectively.

### Proton therapy

Treatment plans for the line-scanning proton therapy were created by Eclipse TPS (Varian Medical Systems, Palo Alto, CA). Proton therapy was performed by a cyclotron (Sumitomo Heavy Industries, Ltd., Tokyo, Japan). Two opposite beams (90 and 270°) with multiple layer scanning capacity were used. All patients received a dose covering 50% volume (D50%) prescription of 63 GyE for PTV in 21 Fr. The proton convolutional superposition was used as the dose calculation algorithm, and the dose grid size was 2.0 mm for all dimensions. The PTV margin was defined as the CTV plus internal margin (IM) plus 3-mm set-up margin (SM). The IM was 5 mm in the anterior direction and 3 mm in other directions. The distal margin was 7 mm beyond the PTV, which was set as 3.5% of the maximum required range distance. The dose constraints of the rectum were V60 Gy <10%, V50 Gy < 20% and V30 Gy <30%. The dose constraints of the rectum were volume covered by 60 Gy (V60 Gy) <10%, V50 Gy < 20% and V30 Gy <30%.

### Evaluation and statistical analysis

First, we performed a global comparison using the areas of the rectum dose–volume histogram (DVH) curve of each patient (area under curve, AUC) as variable [10], and the mean AUCs among the techniques were compared using Wilcoxon’s test. Second, for all treatment plans, the doses received by at least 80, 50, 20 and 2% (D80, D50, D20 and D2% respectively) of the rectum and bladder were calculated, and the dose indices were compared between SO(+) and SO(−) by Wilcoxon’s test. The reduction rate of the rectal dose was calculated using the following equation:

$$ Reduction\ rate\ \left(\%\right)=\left(\frac{D_{post}-{D}_{pre}}{D_{post}}\right)\times 100 $$

*,*where ${D}_{pre}$ was the rectum dose in SO(−) and ${D}_{post}$ corresponded to that in SO(+). Then, we compared the reduction rates between the five techniques by analysis of variance (ANOVA) and Tukey–Kramer tests. All image and dose analyses were performed in MIM Maestro software (Cleveland, OH). All statistical analysis was performed by JMP Pro ver 14.0 (SAS Institute Inc., Cary, NC) with the level of significance at *P* < 0.01.

## RESULTS

This study included 20 patients who underwent prostate radiotherapy with the hydrogel spacer. Considering baseline characteristics, the risk classification was high in 4 cases, intermediate in 14 cases and low in 2 cases. The prostate specific antigen (PSA) was 9.81 ± 4.88 ng/mL, the mean Gleason score was 7.15 ± 1.24 and the mean patient age was 72.6 ± 6.1 years. In this study, the average CTV for all patients was 30.1 ± 11.4 mL. Table 1 shows the PTV dose indices for all treatment techniques. For most of the PTV dose indices, no significant difference was noted between SO(−) and SO(+), while there was a significant difference between some dose indices (*P* < 0.01; VMAT D98% and D95%, and CK D50% and D2%). However, we found that the dose differences themselves were small and had no effect on the PTV dose coverage in a clinical situation.

**Table 1:** Dose index of the PTV for the treatment plans on pre- and post-CT images and the results for the five irradiation techniques

	Absolute dose (mean ± SD, Gy or GyE)											
	D98%			D95%			D50%			D2%		
	SO(−)	SO(+)	*P*-value	SO(−)	SO(+)	*P*-value	SO(−)	SO(+)	*P*-value	SO(−)	SO(+)	*P*-value
3DCRT	71.2 ± 0.8	71.2 ± 1.1	0.7012	72.5 ± 0.5	72.6 ± 0.6	0.4142	75.9 ± 0.3	76.0 ± 0.4	0.041	78.1 ± 0.8	78.6 ± 0.8	0.0484
VMAT	71.6 ± 1.9	73.5 ± 1.6	0.0003	73.5 ± 1.2	74.2 ± 1.1	0.0009	76.1 ± 0.0	76.1 ± 0.1	0.8204	77.5 ± 0.4	77.5 ± 0.4	0.2196
HT	71.6 ± 1.8	72.5 ± 1.5	0.0637	73.8 ± 1.3	74.3 ± 0.8	0.1231	76.4 ± 0.1	76.4 ± 0.1	0.3634	77.8 ± 1.0	77.9 ± 0.8	0.3683
CK	35.5 ± 1.0	35.1 ± 0.4	0.379	36.2 ± 0.2	36.2 ± 0.2	0.8618	39.8 ± 1.2	40.4 ± 0.8	0.0053	45.1 ± 1.6	46.0 ± 1.3	0.0062
Proton	57.9 ± 2.9	58.6 ± 2.5	0.043	59.8 ± 2.1	60.3 ± 1.6	0.0319	63.5 ± 0.3	63.6 ± 0.4	0.9055	65.8 ± 0.4	65.5 ± 0.8	0.4467

**Fig. 1.**
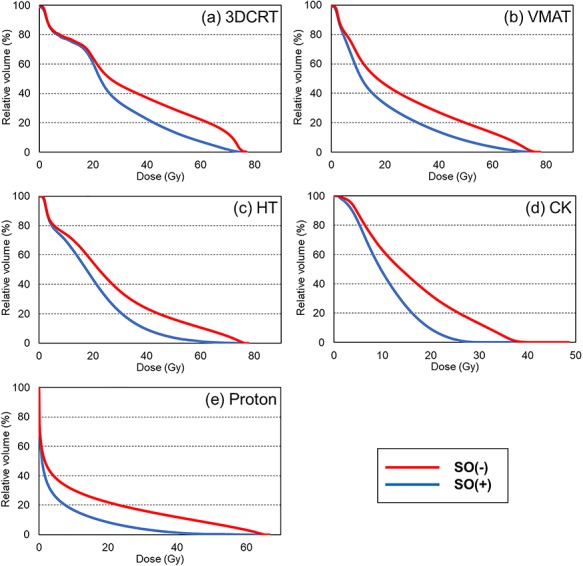
Average dose–volume histogram (DVH) of the rectum in 20 patients and the results of five modalities: (**a**) 3DCRT, (**b**) VMAT, (**c**) HT, (**d**) CK and (**e**) proton. Red and blue solid lines show the result of SO(−) and SO(+), respectively.

**Fig. 2.**
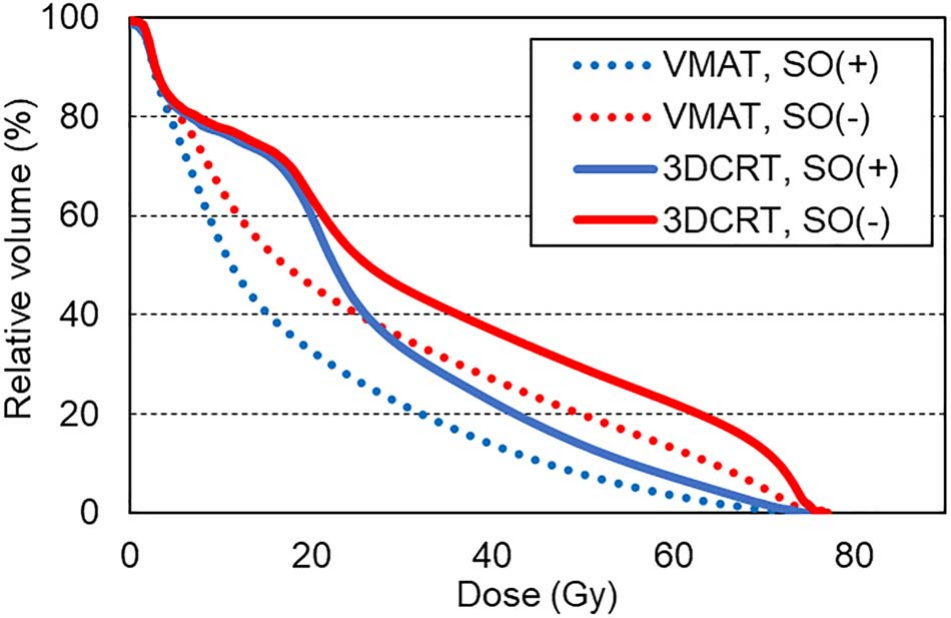
Comparison of DVHs of the rectal dose between 3DCRT and VMAT. Dotted line shows VMAT [red: SO(−), blue: SO(+)] and solid line shows 3DCRT [red, SO(−); blue, SO(+)].

**Fig. 3.**
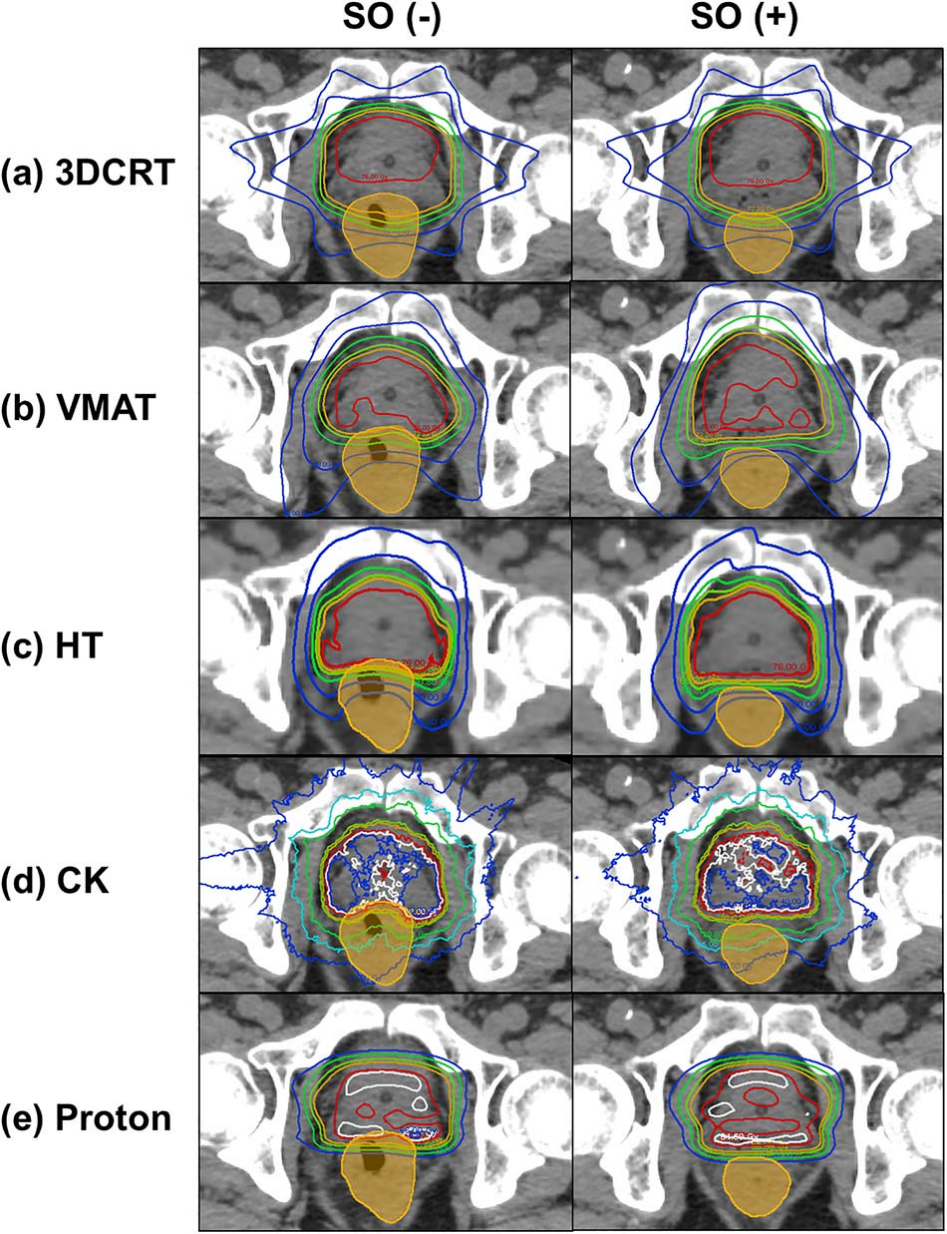
Typical dose distribution of SO(−) and SO(+) and the results of five modalities: (**a**) 3DCRT, (**b**) VMAT, (**c**) HT, (**d**) CK and (**e**) proton. The contour of the orange color illustrates the rectum.

Figure 1 shows the results of the average DVH of the rectum in 20 patients for five modalities: (a) 3DCRT, (b) VMAT, (c) HT, (d) CK and (e) proton. The red and blue solid lines show the results of SO(−) and SO(+), respectively. The dose index of the rectum and bladder for the treatment plans on SO(−) and SO(+) are shown in Tables 2 and 3, respectively. For the rectum, significant differences (*P* < 0.001) in D2%, D20% and D50% were observed for all irradiation techniques. However, D80% did not show significant difference between the treatment plans on SO(−) and SO(+). For the bladder, no significant difference was found between the techniques. From another point of view, Figure 2 shows the comparison of DVHs of rectal dose between 3DCRT and VMAT. Table 4 shows the comparison of the rectal dose between them, and the values of D2% and D20% of 3DCRT [SO(+)] were significantly (*P* < 0.001) lower than that of VMAT [SO(−)]. Furthermore, the areas under the rectum DVH curves were 34.15 ± 3.67 and 34.36 ± 5.24% (*P* = 0.7841) for 3DCRT with hydrogel spacer and VMAT without hydrogel spacer, respectively. For all modalities, typical dose distributions of SO(−) and SO(+) are shown in Figure 3.

**Fig. 4.**
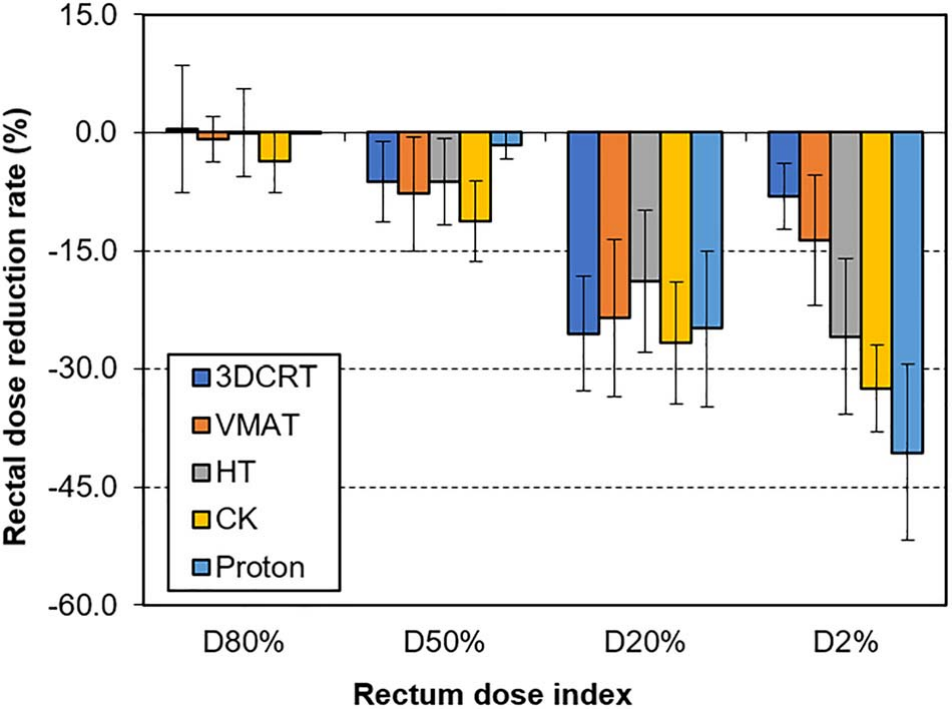
Rectal dose reduction rate using hydrogel spacer in five techniques in 20 patients (average and standard deviation).

Figure 4 shows the rectal dose reduction rate using hydrogel spacer in the five techniques. All rectum dose indices of all techniques trended to reduce by using SO. Except for D80%, significant difference (ANOVA *P*-value < 0.0001) was noted between the five techniques. In particular for D2%, according to the Tukey–Kramer test, the rectal dose changed at −40.61 ± 11.19, −32.44 ± 5.51, −25.90 ± 9.89, −13.63 ± 8.27 and −8.06 ± 4.19%, for proton therapy, CK, HT, VMAT, and 3DCRT, respectively.

Furthermore, the correlation between the effectiveness of using hydrogel spacer and tumor size was evaluated for all treatment techniques. Table 5 shows the Spearman’s rank correlation coefficient between the rectal dose reduction rate and CTV volume. No correlations were found between them for all irradiation techniques.

**Table 2:** Dose index of the rectum for the treatment plans on pre- and post-CT images and the results for the five irradiation techniques

	Absolute dose (mean ± SD, Gy)											
	D80%			D50%			D20%			D2%		
	SO(−)	SO(+)	*P*-value	SO(−)	SO(+)	*P*-value	SO(−)	SO(+)	*P*-value	SO(−)	SO(+)	*P*-value
3DCRT	8.64 ± 5.64	8.98 ± 6.85	0.6742	27.05 ± 5.19	22.29 ± 3.76	<0.0001	61.47 ± 6.92	40.08 ± 6.10	<0.0001	74.26 ± 0.89	68.14 ± 3.19	<0.0001
VMAT	5.66 ± 2.21	4.98 ± 2.11	0.1231	17.27 ± 4.04	11.38 ± 3.75	<0.0001	49.78 ± 5.32	31.90 ± 6.91	<0.0001	72.39 ± 1.70	62.03 ± 7.04	<0.0001
HT	6.67 ± 4.26	6.65 ± 4.69	0.4749	22.21 ± 3.81	17.48 ± 5.17	<0.0001	44.49 ± 5.45	30.15 ± 5.64	<0.0001	73.36 ± 7.83	53.68 ± 7.26	<0.0001
CK	6.71 ± 1.73	5.38 ± 1.86	0.0005	13.87 ± 2.83	9.76 ± 2.13	<0.0001	25.50 ± 3.72	15.83 ± 2.34	<0.0001	36.10 ± 1.52	24.33 ± 1.81	<0.0001
Proton	0.12 ± 0.14	0.09 ± 0.08	0.2699	1.94 ± 1.32	0.91 ± 0.41	<0.0001	23.29 ± 7.15	7.15 ± 2.61	<0.0001	61.35 ± 3.10	35.77 ± 6.51	<0.0001

**Table 3:** Dose index of the bladder for the treatment plans on pre- and post-CT images and the results of five irradiation techniques

	Absolute dose (mean ± SD, Gy)											
	D80%			D50%			D20%			D2%		
	SO(−)	SO(+)	*P*-value	SO(−)	SO(+)	*P*-value	SO(−)	SO(+)	*P*-value	SO(−)	SO(+)	*P*-value
3DCRT	16.02 ± 15.26	15.34 ± 14.70	0.7841	36.80 ± 17.29	34.36 ± 21.74	0.4304	66.01 ± 8.78	63.64 ± 13.44	0.6742	76.72 ± 1.35	77.17 ± 1.25	0.2943
VMAT	18.52 ± 11.55	17.65 ± 12.12	0.2943	41.52 ± 14.22	39.29 ± 17.14	0.3488	69.30 ± 5.52	68.19 ± 10.45	0.8695	76.53 ± 0.38	76.49 ± 0.59	0.8194
HT	15.30 ± 10.99	15.31 ± 11.32	0.6742	38.54 ± 13.22	33.32 ± 16.05	0.0897	65.79 ± 6.61	62.38 ± 11.84	0.2305	76.82 ± 0.27	76.95 ± 0.54	0.4692
CK	13.41 ± 4.74	12.00 ± 5.56	0.114	19.94 ± 5.80	19.21 ± 6.69	0.436	30.85 ± 4.18	29.83 ± 6.03	0.4749	39.87 ± 0.77	39.90 ± 0.89	0.953
Proton	3.17 ± 4.57	2.68 ± 3.04	0.5459	15.55 ± 13.87	16.48 ± 13.87	0.9854	43.97 ± 11.79	43.80 ± 14.67	0.9563	64.71 ± 0.42	64.72 ± 0.55	0.6944

## DISCUSSION

Recently, both conventional treatment in 2 Gy/Fr and SBRT with high dose fraction have been used for localized prostate cancer treatment [11, 12]. The constraints of the rectum dose may be different depending on fractioned doses and total doses; therefore, it should comply with the dose constraints conforming to the fractionation, with or without SO. To the best of our knowledge, no studies have compared various types of external radiotherapy techniques for rectal dose reduction using SpaceOAR™. Therefore, in this study, we compared the efficacy of spacer placement between five irradiation techniques, and some interesting results were obtained. First, the DVH curves of 3DCRT with hydrogel spacer and VMAT without hydrogel spacer were almost the same because of the AUC analysis (Fig. 2 and Table 4). Furthermore, for high- and middle-dose regions (D2% and D20%), the former showed significantly lower rectal dose than the latter. This has not been mentioned in previous reports. Many facilities are still implementing 3DCRT to prostate cancer, because no high-precision machine is available and clinical staff are limited. Our results provide useful evidence with regard to such facilities, indicating that the former technique might be used instead of conventional VMAT as treatment for prostate cancer in such facilities. However, it is noted that VMAT has more benefit than 3DCRT for dose painting in PTV, such as decreasing urethra dose or increasing dose in high-risk regions.

Second, our results showed that the rectal dose reduction in the high-dose region such as D2% depended on the irradiation technique. In particular, proton therapy has Bragg peak that shows potential benefit compared with X-ray therapy; thus, it has high gradient dose distribution and larger rectal dose reduction than other techniques. Furthermore, some previous reports mentioned the safe use of the spacer during proton therapy [13, 14]. Taking into account their results, the use of a hydrogel spacer for proton therapy is highly recommended. In addition, CK showed large dose reduction because it is conventionally used for SBRT, which shows higher dose gradient in the target boundary than conventional radiotherapy. However, although we investigated the techniques in a clinical setting, some facility uses VMAT and HT combined with SBRT, and they were not evaluated in this study.

**Table 4:** Comparison of the rectal doses and AUC between VMAT without hydrogel spacer [SO(−)] and 3DCRT with hydrogel spacer [SO(+)]

	Absolute dose (mean ± SD, Gy)		
	VMAT, SO(−)	3DCRT, SO(+)	*P*-value
D2%	72.39 ± 1.70	68.14 ± 3.19	0.0003
D20%	49.78 ± 5.32	40.08 ± 6.10	0.0003
D50%	17.27 ± 4.04	22.29 ± 3.76	0.0003
D80%	5.66 ± 2.21	8.98 ± 6.85	0.2035
	Area under curve (%)		
AUC	34.15 ± 3.67	34.36 ± 5.24	0.7841

**Table 5:** Spearman’s rank correlation coefficient between the rectal dose reduction rate and CTV volume

	Spearman’s rank correlation coefficient			
	D80%	D50%	D20%	D2%
3DCRT	0.0286	−0.0827	0.1519	0.4256
VMAT	0.1008	−0.0481	−0.2030	−0.1489
HT	0.0045	0.0301	−0.1940	0.1308
CK	0.0090	−0.0632	−0.3182	0.4662
Proton	−0.2248	−0.1669	−0.2977	0.0496

Third, our results showed no correlations between the rectal dose reduction rate and CTV for all irradiation techniques. The reason is that the rectal dose reduction rate depends on the complexity of the shape of the hydrogel spacer rather than the size of CTV. However, only the prostate was defined as CTV in this study; therefore, the relationship of the rectal dose reduction rate with the prostate cancer staging is beyond the scope of this investigation, and a further study is needed.

The effectiveness of using hydrogel spacer could depend on the size of the PTV margin. In this study, the rectal dose reduction with SO was evaluated by using the clinical margin setting for all technologies. However, the size of the PTV margin for prostate radiotherapy could be different between institutions depending on the irradiation technology and presence of image-guided radiotherapy. Therefore, our results could vary depending on the size of the PTV margin, especially the posterior directional expansion. In addition, the rectal dose reduction rate was thought to be defined by the relationship between the PTV margin size and the shape of the hydrogel spacer. Furthermore, by comparing conventional treatment and SBRT, the latter is likely to benefit from hydrogel spacer because of the steep dose gradient. Further study is required to evaluate the effect of different margin sizes for the same irradiation technique.

This work has some limitations. First, the pre- and post-CT images differed slightly from each other because of the bladder volume change at the time of implementation and the displacement of the rectum due to the placement of the hydrogel spacer. Second, some cases were not suitable for bladder dose constraints because the bladder volume was smaller than the one in the clinical situation, while the dose was reduced as much as possible by planners. Third, dose prescription and dose constraints varied among irradiation techniques. Additionally, the beam arrangement of each technique was constant, which might affect the results. However, this condition is often used clinically in our facilities; thus, it is important to show the benefit of the hydrogel spacer in such conditions.

## CONCLUSION

The results of this study demonstrated that all external radiotherapy modalities with hydrogel spacer could reduce the rectal dose. The 3DCRT with hydrogel spacer would reduce medical costs by replacing conventional VMAT without spacer for prostate cancer treatment from the point of view of the rectal dose. For high-dose gradient regions, proton therapy and SBRT with CK show larger rectal dose reduction than other techniques.
